# Behavioral and Engagement Predictors of Arthroplasty Clinic No-Shows: A Calibrated Machine Learning Analysis

**DOI:** 10.1016/j.artd.2026.102096

**Published:** 2026-08-04

**Authors:** Rashed Alananzeh, Kameel Khabaz, Timothy Liu, Nicole J. Newman-Hung, Alexandra I. Stavrakis, Lauren E. Wessel

**Affiliations:** Department of Orthopaedic Surgery, University of California, Los Angeles, Los Angeles, CA, USA

**Keywords:** Arthroplasty, Clinic nonattendance, No-show, Appointment adherence, Machine learning, Social determinants of health

## Abstract

**Background:**

Missed clinic visits in arthroplasty care can impair preoperative optimization, delay surgery, and disrupt follow-up. Drivers of arthroplasty-specific no-shows remain incompletely characterized.

**Methods:**

We extracted 94,723 scheduled arthroplasty appointments at a single tertiary academic center (March 2013–May 2025), retaining each patient's most recent encounter (N = 17,614) for primary analyses. Demographic, socioeconomic, clinical, scheduling, and prior-attendance variables were drawn from the electronic health record. Multivariable logistic regression and isotonic-calibrated machine learning models estimated no-show risk. Discrimination, calibration, and classification were assessed at multiple operating thresholds, and feature contributions were examined via Shapley Additive exPlanations.

**Results:**

The no-show rate was 6.9%. Prior attendance behavior was the strongest predictor: each 10-percentage-point increase in historical no-show proportion conferred ∼26% higher odds (adjusted odds ratio [aOR]: 1.26 per 10 pp; full-range aOR: 9.86; 95% confidence interval: 6.16–15.76; *P* < .001), and appointment confirmation was strongly protective (aOR: 0.24; 95% confidence interval: 0.19–0.29; *P* < .001). Arthroplasty-relevant diagnoses such as osteoarthritis were associated with lower odds, while social determinants of health showed modest but persistent associations with increased risk. Calibrated machine-learning models achieved good discrimination (area under the receiver operating characteristic curve: 0.80) and calibration (Brier skill +0.12); at a capacity-matched threshold flagging the highest-risk 20%, the best-performing model captured ∼60% of no-shows with 82% specificity. Shapley Additive exPlanations highlighted prior no-show history and confirmation as dominant features.

**Conclusions:**

Arthroplasty clinic no-shows reflects engagement patterns, administrative processes, and patient context. Calibrated, interpretable machine-learning models capturing ∼60% of no-shows within the highest-risk 20% of encounters support prospective evaluation for targeted, supportive outreach with equity safeguards.

## Introduction

Missed outpatient clinic appointments are common and reduce the efficiency and timeliness of healthcare [[1], [2], [3], [4]]. Studies report that the rate of missed outpatient appointments varies by patient population and visit type, with an average missed appointment rate reported between 12% and 23% [[1], [3], [4], [5], [6]]. Recurrent nonattendance is correlated with poor clinical outcomes, including increased hospital admissions and overall morbidity and mortality, indicating that the lack of attendance reflects disengagement from longitudinal care [3,7]. No-show behavior is also shaped by structural barriers and broader social determinants of health (SDOH), which influence access, engagement, and continuity of care [[8], [9], [10]]. Interventions that address patients' social and economic needs have shown promise in improving healthcare engagement and utilization patterns [11].

Arthroplasty patients require longitudinal follow-up preoperatively and postoperatively. Missed appointments can delay a patient's ability to undergo surgery, disrupt surveillance for potential complications, and disrupt communication between multi-disciplinary teams. Postoperative complications are particularly concerning, as the prompt detection and treatment of periprosthetic infections are critical for reducing morbidity. Indeed, prior nonattendance has been associated with increased risk of surgical site infection following total knee arthroplasty [12]. Despite this, there is limited research on no-show behaviors in arthroplasty clinics, with previous studies mainly combining arthroplasty patients into larger specialty groups [13,14].

Predictive modeling can be used to identify appointments that are at high risk for nonattendance and develop targeted operational strategies [15]. These interventions include patient reminders, schedule adjustments, and targeted messaging to vulnerable populations [[16], [17], [18]]. Traditional statistical approaches have been supplemented by recent machine learning techniques that demonstrate improved discriminatory abilities in predicting no-shows in outpatient settings; however, concerns regarding the interpretability and bias of machine learning techniques have limited their adoption in routine clinical practice [15,[19], [20], [21]].

In this study, we aimed to identify predictors of appointment no-shows in arthroplasty clinics using both traditional statistical and interpretable machine learning approaches. The primary outcome was appointment-level no-show (vs. attended) at the patient's most recent scheduled arthroplasty clinic encounter. The primary aim was to identify independent predictors of no-show using multivariable logistic regression. Secondary aims were to (i) develop and evaluate isotonic-calibrated machine learning models for no-show risk estimation and (ii) characterize model performance at clinically meaningful operating thresholds (including a capacity-matched threshold for clinic-based outreach). We hypothesized that prior attendance history and appointment engagement would emerge as dominant predictors alongside socioeconomic factors and that interpretable machine learning methods would complement rather than materially outperform traditional regression approaches [22]. To our knowledge, this is among the largest studies to evaluate calibrated, interpretable machine learning prediction of no-shows specifically in arthroplasty clinics.

## Materials and methods

### Study design

We collected a retrospective cohort of all scheduled arthroplasty appointments across multiple clinic locations within a single tertiary academic medical center between March 2013 and May 2025. The cohort spanned preoperative consultations, postoperative follow-ups, and visits from patients who did not proceed to surgery; visit type was not modeled, as the target was no-show at any scheduled visit. Attendance was determined from the final Epic appointment disposition, a standardized closed-list field populated for all 94,723 extracted encounters.

The analytic cohort retained only “completed” and “no show” encounters; “canceled” (n = 33,183), “left without seen” (n = 87), and “arrived” (n = 6) were excluded. Cancellations, the largest excluded category, were treated as a distinct phenomenon from no-shows: they reflect patient- or clinic-initiated rescheduling with advance notice and require different operational responses (rebooking vs proactive engagement). Virtual encounters (telemedicine, n = 854; telephone, n = 362) were also excluded as they were not offered before 2020 and would confound modality with calendar era. Because patients frequently had multiple encounters during the study period, the primary analysis was conducted at the patient level: each patient contributed a single index appointment defined as their most recent scheduled arthroplasty encounter (N = 17,614). Earlier encounters were used to construct each patient's prior attendance history within the health system. The institutional review board (IRB-25-1084) determined that this study met criteria for exemption from further review.

### Data collection and variable definitions

Data were extracted from the Electronic Health Record (EHR) and organized into the following domains: patient demographics, insurance and financial characteristics, clinical diagnoses, SDOH, appointment scheduling characteristics, and prior attendance behavior. Demographic variables included age at appointment, sex, race, ethnicity, marital status, employment status, and need for interpreter services. Insurance variables included payer type (private, Medicare, Medicaid, military, or other), copay amount, and documentation of copay information. Clinical variables included arthroplasty-relevant diagnoses such as osteoarthritis, chronic pain, and other musculoskeletal conditions documented at the time of scheduling. Comorbidity burden was summarized using the Charlson Comorbidity Index [23]. SDOH were captured using International Classification of Disease (ICD)-9/10 supplementary codes (ICD-9-CM V codes; ICD-10-CM Z codes) [24]. These codes are documented across any healthcare encounter within the health system by any clinical team member, not limited to arthroplasty clinic visits. Any SDOH Z-code was documented for 6878 patients (39.1%), concentrated in the economic and societal domains; environmental, educational, and healthcare Z-codes were each documented in under 1%. Because documentation was sparse and known to be inconsistent [24,25], Z-code domains were modeled as binary present/absent indicators.

Appointment-level variables included appointment type, day of week, month of year, and time of day. Scheduling lead time was defined as the number of days between appointment scheduling and the scheduled visit date. Behavioral variables included whether the appointment was confirmed, whether reminder messages were sent, patient new-visit status, and prior attendance history. Prior no-show behavior was quantified as the number and proportion of missed appointments both within arthroplasty clinics and across other outpatient clinics. We also recorded the attendance status of the patient's most recent prior appointment to capture immediate past behavior.

All predictors reflected information available on or before the scheduled appointment. Appointment confirmations were recorded a median of 4 days (interquartile range: 2.4–4.9) before the visit; reminder flags indicated that automated text messages, email notifications, or MyChart portal alerts were dispatched by the institution's standard preappointment scheduling workflow (flags reflect system-attempted delivery, not patient acknowledgment); and prior-attendance variables were derived exclusively from encounters occurring before the index appointment. The operational prediction target is therefore no-show as of the day before the scheduled appointment.

### COVID-19 considerations

Because the study period spans the COVID-19 pandemic, we examined whether era effects required explicit modeling. Annual no-show prevalence during pandemic years (2020–2022: 7.3–8.2%) was comparable to the prepandemic baseline (2017–2019: 6.9–7.8%), suggesting the pandemic did not materially shift attendance patterns. Year of visit was therefore not added as an explicit model covariate; the temporal validation below provides additional assessment of cross-period generalization.

### Data analyses

Univariate analyses assessed associations between candidate predictors and no-show status. Continuous variables were compared using two-sample *t*-tests, while categorical variables were evaluated with chi-square tests. Effect sizes were calculated using Cohen's d for continuous variables, odds ratios (ORs) for binary variables, and Cramér's V for categorical variables. To account for multiple comparisons and minimize type I error, *P* values were adjusted using the Bonferroni correction.

Variables were prioritized for inclusion in the multivariable logistic regression model if they demonstrated a Bonferroni-corrected univariate *P* < .05; multicollinearity was then assessed using variance inflation factors and redundant variables excluded to ensure model stability. A multivariable logistic regression model was then fit to estimate adjusted associations with no-show status. Adjusted ORs (aORs) with 95% confidence intervals (CIs) were reported. Of the 12 candidate predictors, only copay amount had missing values (13.8%, n = 2431); missingness was handled with a binary missingness indicator and multivariable iterative imputation, preserving missingness as an evaluable predictor. With 1209 no-show events and 18 coefficients, the cohort provided approximately 65 events per coefficient, above conventional minimums for prediction-model development.

All analyses were conducted using Python (version 3.13.2).

### Machine learning analysis

Five machine learning models estimated appointment-level no-show risk: L1-regularized logistic regression (least absolute shrinkage and selection operator [LASSO]), k-nearest neighbors, Random Forest, Extreme Gradient Boosting (XGBoost), and CatBoost. Data were repeatedly partitioned using stratified sampling into training (80%) and held-out testing (20%) sets, repeated across 10 random splits to preserve outcome prevalence. Within each split, missing predictor values were also imputed using multivariable iterative imputation, and predictors were standardized using z-score normalization; preprocessing was fit on the training split and applied to the held-out test split to prevent information leakage.

Hyperparameters were tuned using randomized search with 5-fold stratified cross-validation (25 sampled configurations), optimizing the F1 score, and the best-performing model was selected for each split. F1 was selected as the tuning objective; performance is also reported at Youden-optimal and capacity-matched thresholds to reflect realistic deployment use cases.

Hyperparameter grids spanned model-appropriate ranges; full specifications are available from the corresponding author upon request. Class imbalance was addressed via class weighting; resampling approaches (eg, SMOTE) were avoided to preserve the probabilistic interpretation needed for downstream calibration. The same set of 12 candidate predictors entering the multivariable regression model, together with binary missingness indicators where applicable, were used as inputs to the machine learning models. Each best-performing model was wrapped with isotonic probability calibration (CalibratedClassifierCV, 5-fold internal cross-validation) fit on the training split, and calibrated probabilities were evaluated on the held-out test split. Performance was summarized by discrimination (area under the receiver operating characteristic curve [AUROC] and area under the precision–recall curve), calibration (Brier score, Brier skill score, slope, and intercept), and sensitivity/specificity at the 3 thresholds described above.

**Figure 1:**
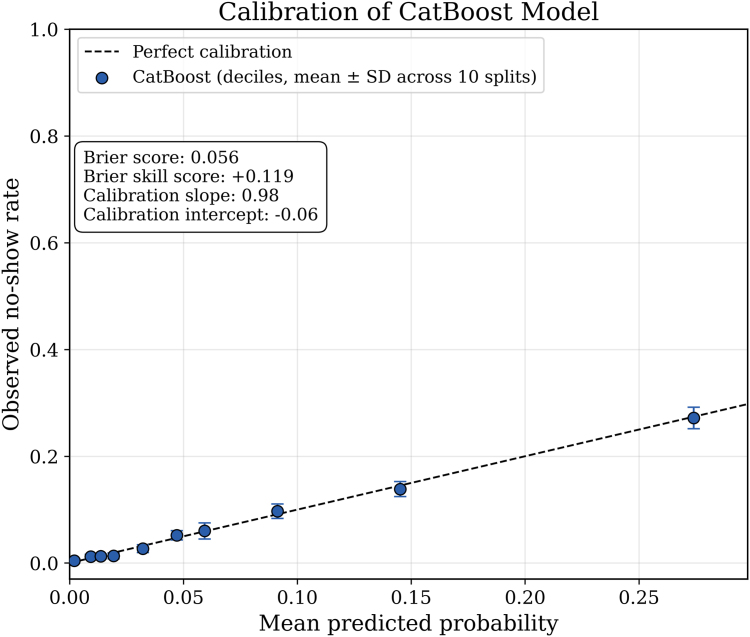
Calibration plot for the CatBoost machine learning model predicting arthroplasty clinic nonattendance. Points represent deciles of predicted probability (mean ± standard deviation across 10 stratified train/test splits); the dashed diagonal represents perfect calibration. Across all deciles of predicted risk, observed no-show rates closely tracked predicted probabilities. Overall calibration was strong (Brier score 0.056; Brier skill score +0.119 relative to a prevalence-based no-information baseline of 0.064; calibration slope 0.98; calibration intercept −0.06). The horizontal axis spans the full range of calibrated predicted probabilities (max ∼0.30), reflecting the empirical ceiling on individual no-show risk after calibration in this rare-outcome (6.86% prevalence) setting.

Reporting followed the TRIPOD + artificial intelligence (AI) guidelines [26]; no external validation cohort was available. Model calibration was visualized as a decile-binned plot for the CatBoost model (Fig. 1). Feature importance was assessed using Shapley Additive exPlanations (SHAP) values, which quantify each predictor's marginal contribution to individual predictions [27].

### Temporal validation

To assess generalization across the study timeline, we performed a temporal hold-out validation in addition to the repeated random-split analysis. The cohort was sorted by appointment date and split at January 1, 2024, yielding a training set of 13,122 encounters from March 2013 through December 2023 (no-show prevalence 7.5%) and a held-out test set of 4492 encounters from January 2024 through May 2025 (no-show prevalence 5.1%). All 5 models were retrained on the temporal training set using the same preprocessing pipeline, hyperparameter search, and isotonic calibration described above. Performance was evaluated on the temporal test set using the same metrics.

### Study population

The analytic cohort comprised 17,614 patients (16,405 attended; 1209 no-shows; no-show rate 6.86%). The mean age was 67.9 y (standard deviation: 14.2), and 59.1% were female; 64.1% were non-Hispanic White, 7.7% Black or African American, 6.1% Asian, and 9.3% Hispanic or Latino. Insurance was private (54.9%), Medicare (25.1%), or Medicaid (12.7%); 5.8% required interpreter services. Full baseline characteristics stratified by attendance are presented in Table 1.

**Table 1:** Patient characteristics and univariate associations with appointment no-show.

Category	Variable	Attended (N = 16,405)	No-show (N = 1209)	Effect size (95% CI)	*P* value (corrected)
Demographics	Age (y)	68.0 (14.0)	66.5 (16.9)	*d* = −0.108	0.046
	Sex Female Male	9727 (59.3%) 6676 (40.7%)	688 (56.9%) 521 (43.1%)	OR 0.910 (0.80, 1.02) Reference	1 —
	Marital status Married Single/other	8463 (51.6%) 7915 (48.2%)	461 (38.1%) 743 (61.5%)	OR 0.61 (0.54–0.69) Reference	<0.001 —
Race/ethnicity	Race identification White Black Asian Other Ethnicity Hispanic/Latino	10,653 (64.9%) 1226 (7.5%) 1027 (6.3%) 1172 (7.1%) 1886 (11.5%)	642 (53.1%) 134 (11.1%) 46 (3.8%) 146 (12.1%) 181 (15.0%)	Reference OR 1.543 (1.28, 1.86) OR 0.592 (0.44, 0.80) OR 1.785 (1.49, 2.14) OR 1.46 (1.24–1.73)	— 0.001 0.106 <0.001 0.002
Financial	Copay amount ($) Missing copay Insurance Private Medicaid Medicare	$7.46 (17.9) 2110 (12.9%) 9161 (55.8%) 1924 (11.7%) 4119 (25.1%)	$1.31 (7.5) 321 (26.6%) 507 (41.9%) 318 (26.3%) 295 (24.4%)	*d =* −0.352 OR 2.44 (2.1–2.8) OR 0.57 (0.51–0.64) OR 2.686 (2.34, 3.08) OR 0.96 (0.84–1.10)	<0.001 <0.001 <0.001 <0.001 1.000
Social	Environmental SDOH Language Interpreter needed Employment Disabled	141 (0.9%) 916 (5.7%) 893 (5.4%)	23 (1.9%) 109 (9.0%) 147 (12.2%)	OR 2.24 (1.4–3.5) OR 1.73 (1.4–2.1) OR 2.40 (1.99, 2.89)	0.001 <0.001 <0.001
Clinical diagnoses	OA Chronic pain Dementia Acute hip fracture Stroke	9644 (58.8%) 11,274 (68.7%) 901 (5.5%) 1016 (6.2%) 338 (2.1%)	539 (44.6%) 649 (53.7%) 121 (10.0%) 140 (11.6%) 47 (3.9%)	OR 0.56 (0.50, 0.63) OR 0.53 (0.47, 0.59) OR 1.91 (1.57, 2.34) OR 1.98 (1.65, 2.39) OR 1.92 (1.41, 2.62)	<0.001 <0.001 <0.001 <0.001 0.006
Scheduling and behavior	Logistics Lead time (d) Days since last visit Engagement Confirmed Appt MyChart Reminder Text Reminder	45.29 (61.9) 205.30 (386.5) 5305 (32.3%) 6842 (41.7%) 6405 (39.0%)	51.08 (57.9) 142.31 (290.2) 101 (8.4%) 301 (24.9%) 353 (29.2%)	*d* = 0.094 (0.04, 0.15) *d* = −0.165 (−0.2, −0.1) OR 0.191 (0.16–0.23) OR 0.463 (0.41–0.53) OR 0.644 (0.57–0.73)	0.240 <0.001 <0.001 <0.001 <0.001
History	Last arthroplasty clinic no-show Prior no-show rate (other clinics) Prior no-show rate (arthroplasty clinic)	415 (2.5%) 0.04 (0.09) 0.02 (0.11)	103 (8.5%) 0.09 (0.15) 0.08 (0.22)	OR 3.588 (2.87–4.49) *d* = 0.526 (0.47, 0.59) *d* = 0.441 (0.38, 0.50)	<0.001 <0.001 <0.001

OA, osteoarthritis.

Total patient population (most recent scheduled encounter per patient): N = 17,614. Categorical variables are presented as n (%) of the outcome group column; continuous variables (Age, Copay, Days since last visit, Prior no-show rates, Lead time) are presented as mean (standard deviation). *P* values for categorical variables were determined via chi-square test, while *P* values for continuous variables were determined via independent samples *t*-test. To minimize type I error, all *P* values have been adjusted using the Bonferroni correction for multiple comparisons (N = 187 tests). Effect sizes are reported as odds ratios (OR) for categorical variables and Cohen’s d for continuous variables; Cramér’s V is reported for multicategory nominal variables (eg, day of week). For categorical variables, the baseline comparison group is designated as “Reference.”

## Results

Univariate associations are summarized in Table 1; findings with the largest effect sizes are highlighted below.

### Univariate analysis

#### Socioeconomic variables

Insurance and financial characteristics demonstrated clinically meaningful associations with appointment attendance. Medicaid insurance was associated with markedly higher odds of no-show (OR: 2.69; 95% CI: 2.34–3.08; *P* < .001), whereas private insurance appeared protective (OR: 0.57; 95% CI: 0.51–0.64; *P* < .001). Higher copay amounts were associated with lower odds of no-show (d = −0.352; *P <* .001), while missing copay information was associated with substantially increased no-show risk (OR: 2.44: 95% CI: 2.10–2.80; *P* < .001).

#### Clinical variables

Clinical diagnoses linked to primary arthroplasty indications such as osteoarthritis (OR: 0.56; 95% CI: 0.50–0.63; *P* < .001) and chronic pain (OR: 0.53; 95% CI: 0.47–0.59; *P* < .001) were associated with lower odds of no-show. In contrast, neurological and acute mobility-limiting comorbidities, such as dementia (OR: 1.91; 95% CI: 1.57–2.34; *P* < .001), stroke (OR: 1.92; 95% CI: 1.41–2.62; *P* = .006), and acute hip fractures (OR: 1.98; 95% CI: 1.65–2.39; *P* < .001) were associated with an increased risk of no-show.

#### Behavioral and scheduling-related variables

Scheduling and behavioral variables demonstrated the largest univariate effect sizes among all predictors. Engagement markers were associated with substantially lower odds of no-show, including active appointment confirmation (OR: 0.19; 95% CI: 0.16–0.23; *P* < .001) and receipt of MyChart portal reminders (OR: 0.46; 95% CI: 0.41–0.53; *P* < .001). Prior attendance behavior emerged as a dominant risk factor; patients who missed their most recent arthroplasty appointment had markedly higher odds of a subsequent no-show (OR: 3.59; 95% CI: 2.87–4.49; *P* < .001). A patient’s historical proportion of no-shows across other clinics (d = 0.526; *P <* .001) was among the strongest predictors of no-show. Scheduling lead time differed only modestly between groups (d = 0.094, *P =* .0016) and did not remain significant after Bonferroni correction (*P =* .240).

### Multivariate analysis

**Figure 2:**
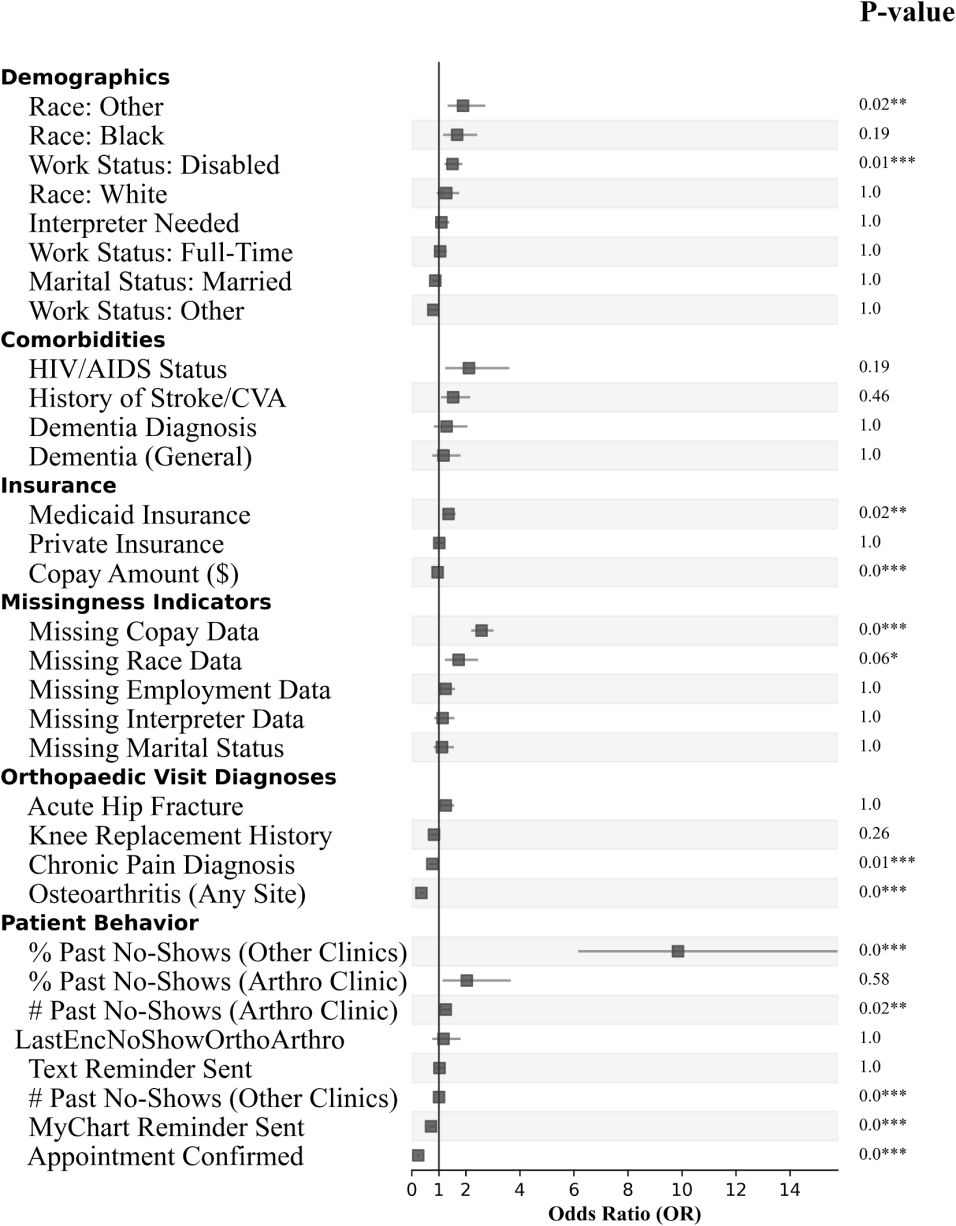
Multivariate analysis results. Forest plot displaying adjusted odds ratios (aORs) with 95% confidence intervals from multivariable logistic regression modeling predictors of appointment nonattendance (N = 17,614). The vertical dashed line at aOR = 1.0 represents the null effect. Points to the right of this line indicate increased odds of nonattendance; points to the left indicate decreased odds (protective association). Error bars represent 95% confidence intervals. Variables are grouped by domain: demographic characteristics, insurance and financial factors, clinical diagnoses, social determinants of health, and behavioral/scheduling factors. Asterisks denote statistical significance after Bonferroni correction (∗*P* < .05; ∗∗*P* < .01; ∗∗∗*P* < .001). Abbreviations: CVA, cerebrovascular accident; OA, osteoarthritis.

In the multivariable logistic regression model adjusting for demographic characteristics, insurance and financial variables, comorbidities, SDOH, patient behavior, and scheduling factors, several predictors remained independently associated with appointment no-show (Fig. 2).

#### Socioeconomic variables

Medicaid insurance remained associated with higher odds of no-show (aOR: 1.36; 95% CI: 1.14–1.63; corrected *P* = .021), and disability-related employment status also remained independently associated with increased no-show risk (aOR: 1.51; 95% CI: 1.22–1.88; corrected *P* < .001). Documentation missingness indicators were strongly predictive: missing copay documentation was associated with increased odds of no-show (aOR: 2.58; 95% CI: 2.20–3.03; *P* < .001), while copay amount was modeled as a continuous predictor and showed a protective association, with the aOR reflecting the change in odds of no-show per $1 increase in copay (aOR: 0.96; 95% CI: 0.95–0.97; *P* < .001). Race effects were more limited after correction; patients categorized as “other” race had higher odds of no-show (aOR: 1.90; 95% CI: 1.32–2.72; corrected *P* = .016), whereas missing race documentation did not meet the corrected significance threshold (corrected *P* = .057).

#### Clinical variables

Selected clinical diagnoses remained independently associated with attendance. Osteoarthritis was strongly protective (aOR: 0.36; 95% CI: 0.30–0.42; *P* < .001), and chronic pain remained protective after adjustment (aOR: 0.76; 95% CI: 0.66–0.88; *P* < .001).

#### Behavioral and scheduling-related variables

Patient behavior and engagement were the strongest independent predictors. A patient's historical percentage of no-shows across other outpatient clinics was strongly associated with no-show: each 10-percentage-point increase in this proportion was associated with approximately 26% higher odds of no-show (aOR: 1.26 per 10-percentage-point increase, full-range aOR: 9.86 for a 0 to 1 change, 95% CI: 6.16–15.76; *P* < .001). Conversely, active engagement through appointment confirmation was strongly protective (aOR: 0.24; 95% CI: 0.19–0.29; *P* < .001). Additional engagement and behavioral history measures remained independently associated with no-show, including receipt of MyChart reminders (aOR: 0.72; 95% CI: 0.61–0.84; Bonferroni-corrected *P* = .0013), the number of prior no-shows within arthroplasty clinics (aOR: 1.26 per prior no-show; 95% CI: 1.11–1.43; corrected *P* = .016), and the number of prior no-shows across other outpatient clinics (aOR: 1.01 per prior no-show; 95% CI: 1.01–1.02; corrected *P* = .0043).

### Machine learning model performance

Discrimination was consistent across models and within split-to-split variability (Table 2). The 4 main models (LASSO, Random Forest, XGBoost, and CatBoost) achieved nearly identical performance (AUROC: 0.80–0.81), with CatBoost marginally best (0.810 ± 0.013). After isotonic calibration, all 4 main models produced probability estimates better than a prevalence-based null (Brier 0.056–0.057 vs 0.064 baseline; Brier skill +0.10 to +0.12) with calibration slopes near 1.0 and intercepts near zero, indicating that predicted probabilities corresponded closely to observed event rates. A decile-binned calibration plot for the CatBoost model confirmed close agreement between predicted probabilities and observed no-show rates across the range of calibrated risk estimates (Fig. 1).

**Table 2:** Machine learning model performance for no-show prediction in arthroplasty clinic.

Model	AUROC	AUPRC	Brier	Brier skill	Cal. Slope	Sens./Spec. (Youden)	Sens./Spec. (Top-20%)
LASSO (L1-logistic)	0.801 ± 0.013	0.253 ± 0.021	0.057 ± 0.001	+0.105 ± 0.014	0.94 ± 0.09	0.75 / 0.71	0.58 / 0.83
Random Forest	0.805 ± 0.014	0.274 ± 0.020	0.056 ± 0.001	+0.119 ± 0.013	0.98 ± 0.10	0.72 / 0.76	0.61 / 0.83
XGBoost	0.806 ± 0.013	0.277 ± 0.018	0.056 ± 0.001	+0.117 ± 0.012	1.00 ± 0.07	0.74 / 0.73	0.59 / 0.83
CatBoost	0.810 ± 0.013	0.277 ± 0.023	0.056 ± 0.001	+0.119 ± 0.015	0.98 ± 0.09	0.76 / 0.72	0.61 / 0.82
k-Nearest Neighbors	0.672 ± 0.022	0.168 ± 0.023	0.061 ± 0.001	+0.044 ± 0.009	1.28 ± 0.16	0.54 / 0.78	0.54 / 0.72

AUPRC, area under the precision–recall curve; Sens., sensitivity; Spec., specificity; SD, standard deviation.

All models were wrapped with isotonic probability calibration (CalibratedClassifierCV, 5-fold) fit on the training split. Brier skill score is computed relative to a no-information baseline of always predicting outcome prevalence (Brier_ref = 0.064); positive values indicate better probability accuracy than the baseline. Calibration slope is estimated from a logistic regression of the observed outcome on the logit of predicted probability (ideal value = 1.0). Two operationally meaningful thresholds are reported: the Youden-optimal threshold maximizes the sum of sensitivity and specificity minus 1, and the "top-20%" threshold flags the highest-risk 20% of test-set encounters, reflecting realistic clinic-outreach capacity. Values reported as mean ± SD across 10 stratified train/test splits.

Because calibrated probabilities for a 6.86% outcome rarely exceed the default 0.5 threshold, classification sensitivity at that cutoff was limited (<0.10) despite very high specificity. At the Youden-optimal operating point, sensitivity exceeded 0.72 with specificity above 0.70. At a capacity-matched threshold flagging the highest-risk 20% of encounters (a realistic scope for clinic-based outreach), all 4 main models captured 58–61% of eventual no-shows with approximately 83% specificity (CatBoost: sensitivity 0.605 ± 0.031, specificity 0.824 ± 0.009). At this threshold, the positive predictive value was approximately 20% (precision 0.202 ± 0.011), roughly a 3-fold enrichment over the cohort base rate of 6.86%, corresponding to approximately 5 flagged appointments per captured no-show.

#### Temporal validation

In the temporal validation, the 4 main models retained substantial discriminative ability on the 2024–2025 held-out test set (AUROC 0.77 for LASSO, Random Forest, XGBoost, and CatBoost; AUROC 0.71 for k-nearest neighbors; Table 3). At the capacity-matched threshold flagging the highest-risk 20% of test-set encounters, CatBoost captured 60% of eventual no-shows with 80% specificity, and the 4 main models collectively captured 58–61% of no-shows. Calibration slope was modestly attenuated (∼0.5 for the main models), reflecting the lower no-show base rate in the test period (5.1% vs 7.5% in training); this would be addressed by periodic recalibration in any prospective deployment.

**Table 3:** Machine learning model performance on temporal validation.

Model	AUROC	AUPRC	Brier	Brier skill	Cal. Slope	Sens./Spec. (Youden)	Sens./Spec. (Top-20%)
LASSO (L1-logistic)	0.765	0.173	0.063	−0.30	0.47	0.77 / 0.65	0.58 / 0.82
Random Forest	0.767	0.174	0.062	−0.27	0.48	0.73 / 0.71	0.59 / 0.79
XGBoost	0.771	0.184	0.061	−0.25	0.54	0.73 / 0.72	0.61 / 0.82
CatBoost	0.769	0.186	0.060	−0.23	0.45	0.71 / 0.72	0.60 / 0.80
k-Nearest Neighbors	0.710	0.130	0.051	−0.04	0.93	0.71 / 0.66	0.44 / 0.81

AUPRC, area under the precision–recall curve; Sens., sensitivity; Spec., specificity.

Training set: n = 13,122 encounters (March 2013 through December 2023; no-show prevalence 7.5%). Test set: n = 4492 encounters (January 2024 through May 2025; no-show prevalence 5.1%). All models were retrained on the temporal training set and wrapped with isotonic probability calibration (CalibratedClassifierCV, 5-fold internal cross-validation). The negative Brier skill scores reflect the lower no-show base rate in the test period compared to training (5.1% vs 7.5%), to which the isotonic calibrator was fit; this calibration shift would be addressed by periodic recalibration in prospective deployment. The "top-20%" threshold flags the highest-risk 20% of test-set encounters.

### Model interpretability and feature contributions

**Figure 3:**
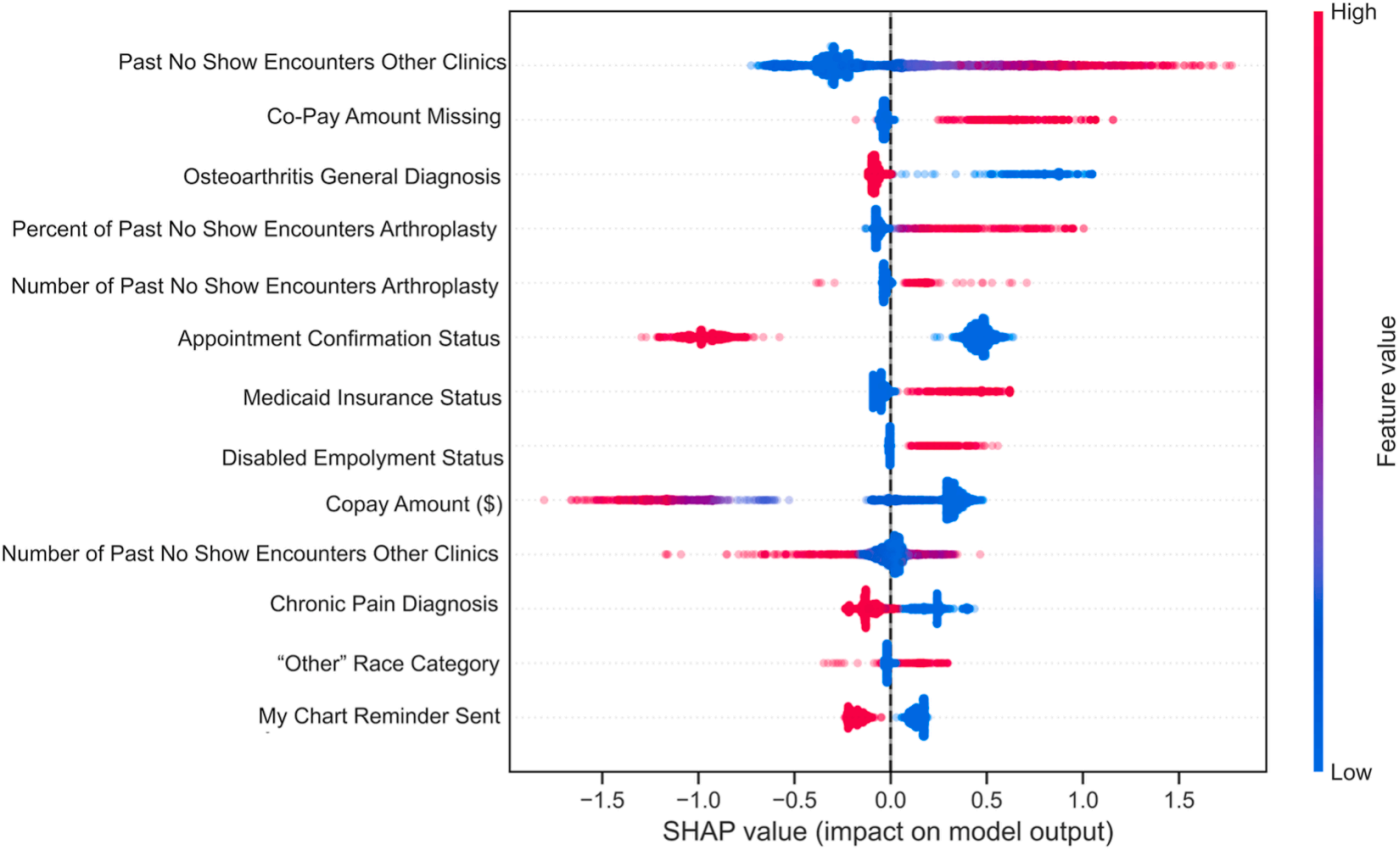
SHAP summary plot derived from the CatBoost model. Shapley Additive exPlanations (SHAP) summary plot illustrating the relative importance and directionality of features contributing to appointment nonattendance. Each point represents an individual visit, with color indicating feature value (red = higher value, blue = lower value). Positive SHAP values indicate increased predicted probability of no-show, while negative values indicate reduced risk.

To interpret model predictions, SHAP values were computed for the final CatBoost model (Fig. 3). SHAP summary plots were used to rank predictors by their mean absolute contribution and to characterize the direction of their association with predicted no-show. Higher historical no-show burden, particularly the percentage of prior no-shows across other outpatient clinics and within arthroplasty clinics, consistently increased predicted no-show risk.

In contrast, appointment confirmation was strongly protective. Several socioeconomic and documentation-related factors also contributed to risk, including missing copay documentation, Medicaid insurance, and disability-related employment status. Clinical factors related to arthroplasty indications, including osteoarthritis and chronic pain, showed protective contributions in the model.

## Discussion

This large retrospective cohort study of arthroplasty clinic patients identified consistent predictors of appointment no-shows across univariate, multivariable regression, and machine learning analyses. Prior attendance behavior and engagement markers, particularly historical no-show burden and appointment confirmation, emerged as the strongest predictors. Demographic and socioeconomic factors retained independent associations after adjustment, indicating that no-show risk reflects both patient context and engagement. This finding is consistent with prior outpatient literature identifying prior no-show history as a reproducible predictor of future missed visits [1,[28], [29], [30]]. Overall, these findings support targeted operational interventions, such as strengthened confirmation workflows and proactive outreach to patients with high prior no-show burden, while remaining attentive to persistent socioeconomic and documentation-related barriers.

Across analytical approaches, the most prominent signal was historical no-show burden within the same health system, particularly the percentage of prior missed encounters in other outpatient clinics. In the adjusted model, this measure remained strongly associated with arthroplasty clinic no-shows. Conceptually, prior attendance behavior likely aggregates multiple barriers that are incompletely captured in structured EHR fields, including transportation challenges, competing responsibilities, and friction in scheduling or registration workflows [9,10,[31], [32], [33], [34], [35], [36], [37]].

Our findings align with arthroplasty-specific literature demonstrating similar patterns where Curry et al. reported a 9.3% no-show rate in an urban joint arthroplasty clinic, with government insurance, younger age, and Black/Hispanic race associated with increased nonattendance [13]. However, our data extend beyond demographic and socioeconomic variables to highlight engagement-related targets (appointment confirmation, prior attendance history) that could be tested prospectively for intervention value. Our observed no-show rate of 6.86% was lower than many reported arthroplasty cohorts, potentially reflecting differences in patient populations, scheduling infrastructure, or engagement strategies [13,28,38]. Our work extends recent orthopaedic prediction-modeling efforts [39] by focusing on arthroplasty, employing isotonic calibration for interpretable probability estimates, and integrating equity-aware deployment considerations.

Appointment confirmation was consistently protective in both regression and SHAP analyses, consistent with literature on reminder and confirmation interventions [17,[40], [41], [42], [43]], and predictive-model-driven outreach has shown additional benefit in randomized trials [15]. Because this was an observational study, confirmation should not be interpreted as causal and may partly reflect pre-existing intent to attend. Nonetheless, the strong association identifies confirmation workflows as a practical target for prospective testing, potentially with stepped reminders or navigation support for higher-risk appointments [[40], [41], [42], [43]].

Socioeconomic and documentation-related variables demonstrated meaningful independent associations. Medicaid coverage and disability-related employment status remained associated with increased no-show risk after adjustment, consistent with broader evidence that structural and socioeconomic barriers shape access and continuity of care [9,10,[33], [34], [35], [36], [37]]. Prior work has similarly shown that unmet social needs and neighborhood-level factors contribute meaningfully to missed appointments even after controlling for individual patient characteristics [32,33]. Documentation completeness also carried signal: missing copay documentation and missing race documentation were both associated with no-show in adjusted models suggesting that registration or documentation processes may themselves identify higher-risk encounters, though missingness may be nonrandom and reflect administrative workflows rather than patient characteristics.

Consistent with our prestated hypothesis [22], machine learning models did not materially outperform multivariable logistic regression for discrimination; both approaches identified the same dominant predictors. The added value of the machine learning pipeline lay in 3 deployment-oriented properties: (i) isotonic calibration produced probability estimates with slopes near 1.0 and Brier skill +0.10 to +0.12, enabling calibrated risk for capacity-matched outreach; (ii) SHAP attribution provided patient-level explanation (Fig. 3), complementing aggregate regression coefficients; and (iii) tree-based ensembles offered marginally better calibration (∼1.00 vs 0.94 for LASSO) and a modest gain in average precision (∼0.025). This pattern is consistent with systematic reviews showing that feature quality and operational integration often matter more than algorithmic complexity in EHR prediction tasks [39, 44, 45].

Temporal validation supported the proposed prospective use case: ranking-based performance was preserved on the 2024–2025 test set, with capacity-matched sensitivity essentially unchanged (60% of no-shows in the top 20% of predicted risk). Calibration was modestly attenuated due to the lower base rate in the test period, supporting periodic recalibration as a routine deployment step.

Finally, these results carry equity implications that are directly relevant to operational deployment. Predictive tools that incorporate prior attendance and administrative engagement may inadvertently encode structural disadvantage; if used to restrict scheduling access or deprioritize care, such tools could worsen disparities. A more appropriate application is to use predicted risk to allocate supportive resources (eg, higher-touch reminders, care navigation, transportation assistance, flexible scheduling) with ongoing monitoring for differential performance across subgroups and explicit safeguards against punitive use [40,[46], [47], [48], [49], [50], [51], [52]]. Tarabichi et al. demonstrated that predictive model-driven telephone outreach to high-risk patients reduced overall no-show rates and specifically reduced racial disparities, predominantly by improving access for Black patients [46].

### Limitations

This study has several limitations that warrant consideration. The cohort was derived from a single tertiary academic health system, which may limit generalizability to settings with different patient populations or scheduling infrastructures. The observational design precludes causal inference; associations between engagement markers (eg, confirmation status), financial documentation, and no-show may reflect unmeasured confounding. Additionally, using each patient's most recent scheduled encounter as the index visit may introduce survivorship-style selection bias, as patients who remained engaged long enough to accumulate encounters may differ systematically from those who disengaged earlier. The analysis focused exclusively on no-shows; late cancellations that prevent slot refill represent a related operational problem not addressed by this work.

Reliance on structured EHR fields limited measurement of contextual determinants of attendance, including transportation availability, employment flexibility, caregiving responsibilities, and health literacy, all of which may influence appointment adherence [9,10,[32], [33], [34], [35], [36], [37]]. Prior attendance behavior was measured only within this health system and may not reflect appointments attended or missed outside the system.

SDOH were captured using ICD-9 V-codes and ICD-10 Z-codes, which are known to be underutilized and inconsistently documented; therefore, a value of "0" should be interpreted as no documented code rather than confirmed absence of social risk [24,25]. Missingness in administrative and financial fields may be nonrandom (eg, missing copay documentation may reflect workflow or registration differences), and missingness itself may function as a marker of administrative engagement.

Although a temporal hold-out validation supported short-horizon generalization, longer-horizon performance under sustained operational change remains an open question, and external validation in different health systems will be necessary before broader implementation [19,21].

## Conclusions

Prior attendance behavior and appointment engagement, together with selected demographic, socioeconomic, and clinical variables, were the most consistent predictors of no-show across regression and machine learning approaches. Calibrated, interpretable machine learning models captured approximately 60% of no-shows within the highest-risk 20% of encounters. These findings support prospective evaluation of targeted, supportive outreach (eg, optimized confirmation workflows, stepped engagement) paired with equity safeguards to ensure prediction increases support rather than restricting access [[40], [41], [42], [43],^46^,^49^].

## Data and code availability

Analysis code is available from the corresponding author upon reasonable request. Patient-level data cannot be shared publicly due to HIPAA and institutional privacy restrictions; deidentified aggregate data are available on request.

## Conflicts of interest

The authors declare there are no conflicts of interest.

For full disclosure statements refer to https://doi.org/10.1016/j.artd.2026.102096.

## CRediT authorship contribution statement

**Rashed Alananzeh:** Writing – review & editing, Writing – original draft, Validation, Software, Methodology, Investigation, Formal analysis, Data curation, Conceptualization. **Kameel Khabaz:** Writing – review & editing, Validation, Software, Methodology, Investigation, Formal analysis, Data curation. **Timothy Liu:** Writing – review & editing, Investigation, Data curation. **Nicole J. Newman-Hung:** Writing – review & editing, Supervision, Methodology, Investigation, Conceptualization. **Alexandra I. Stavrakis:** Writing – review & editing, Supervision, Resources. **Lauren E. Wessel:** Writing – review & editing, Validation, Supervision, Resources, Project administration, Methodology, Investigation, Conceptualization.

## Appendix A Supplementary data

Conflict of Interest Statement for Stavrakis

Conflict of Interest Statement for Khabaz

Conflict of Interest Statement for Wessel

Conflict of Interest Statement for Newman-Hung

Conflict of Interest Statement for Alananzeh

Conflict of Interest Statement for Liu
